# Speciation across the Earth driven by global cooling in terrestrial orchids

**DOI:** 10.1073/pnas.2102408120

**Published:** 2023-07-10

**Authors:** Jamie B. Thompson, Katie E. Davis, Harry O. Dodd, Matthew A. Wills, Nicholas K. Priest

**Affiliations:** ^a^The Milner Centre for Evolution, Department of Life Sciences, University of Bath, Bath BA2 7AY, United Kingdom; ^b^Department of Biology, University of York, York YO10 5DD, United Kingdom

**Keywords:** biodiversity, macroevolution, climate change, biogeography, diversification

## Abstract

Although climate change has been implicated as a major catalyst of diversification, its effects are thought to be inconsistent and much less pervasive than localized climate or the accumulation of species with time. Focused analyses of highly speciose clades are needed in order to disentangle the consequences of climate change, geography, and time. Here, we show that global cooling shapes the biodiversity of terrestrial orchids. Using a phylogeny of 1,475 species of Orchidoideae, the largest terrestrial orchid subfamily, we find that speciation rate is dependent on historic global cooling, not time, tropical distributions, elevation, variation in chromosome number, or other types of historic climate change. Relative to the gradual accumulation of species with time, models specifying speciation driven by historic global cooling are over 700 times more likely. Evidence ratios estimated for 212 other plant and animal groups reveal that terrestrial orchids represent one of the best-supported cases of temperature-spurred speciation yet reported. Employing >2.5 million georeferenced records, we find that global cooling drove contemporaneous diversification in each of the seven major orchid bioregions of the Earth. With current emphasis on understanding and predicting the immediate impacts of global warming, our study provides a clear case study of the long-term impacts of global climate change on biodiversity.

## Significance

The staggering biodiversity of angiosperms has been difficult to reconcile with the gradual Darwinian process thought to create it. Changes in climate through the Earth’s history could have instigated this diversification, but perceived variability across clades and geography has restrained generalization. In this paper, we reconstruct the evolutionary history of a rich terrestrial orchid subfamily studied by Darwin (Orchidoideae, ~5,000 species) and use >2.5 million georeferenced records to test how and where those orchid species arose. We find that global cooling between the Miocene and present day spurred terrestrial orchid speciation across the Earth. This work resolves orchidoid phylogeny and provides a striking example of how historic climate change drives global patterns of biodiversity.

Charles Darwin’s “abominable mystery” was why diversification can happen so rapidly (1, 2). The abrupt appearance of diverse clades of angiosperms was a challenge not only to Darwin’s theory, but to evolution itself (3). Under mounting pressure from paleobotanists and other groups, Darwin asserted an admittedly “wretchedly poor conjecture” that the patterns could be explained by an ancient origin of angiosperms on a “small isolated continent in the southern hemisphere” (3, letter to Joseph Hooker, 22 July 1879, Darwin Correspondence Project). Since then, there has been active inquiry into the forces determining distribution, geographic origins, and timing of diversification of all angiosperm clades (3–5).

A growing body of theory argues that the origins and contemporary distributions of biodiversity are largely determined by historic climate change (6, 7). The problem has been, however, that there are few clear supporting examples. The consequences of climate change on diversification are generally thought to be inconsistent, varying between closely related groups of taxa (8, 9), the type of climate change (10), and the ecoregion in which it occurs (11, 12). In contrast, a substantial proportion of biodiversity can be explained by the gradual accumulation of species over time (13), together with the influences of geographic factors including latitude (14, 15) and elevation (16, 17).

In many of the classic cases of adaptive radiation, such as cichlid fish, Darwin’s finches, and *Anolis* lizards, diversification occurred at localized scales. This makes it difficult to determine whether global climate change contributed to diversification. Still, the latitudinal species gradient may not be caused by rapid speciation, as previously thought (14, 15). Global warming remains the most parsimonious temporal climatic explanation for diversification in many animal taxa (13). However, the ability of animals to rapidly shift their distributions according to prevailing ecological conditions makes it difficult to factor out the influence of localized geography. The critical, unresolved question is whether historic climate change can have consistent, global influences on the origins and distributions of organismal biodiversity (9, 18). Cosmopolitan plant clades therefore offer the best model systems in which to disentangle the influences of climate change, geography, and the time of clade origins upon patterns of species richness.

The terrestrial orchid subfamily Orchidoideae (orchidoid orchids) are an ideal group in which to address this question. They exhibit an intriguing combination of recent origins [stem age c.64 Mya (19)] and extraordinary diversity, with 5,000 extant species (20). Their global biogeographical distributions have been recorded in intimate detail. The timing of speciation events relative to other orders of monocots is well established, despite a patchy fossil record (19). Geographic consistency can be established by testing for similarities in responses to climate change in taxa endemic to each of the seven major orchid bioregions (defined by Givnish et al. 21). Moreover, Orchidoideae encompass minimal variation in traits previously associated with speciation, including pollinia, and they lack epiphytism, which contributes to accelerated speciation across all major clades of orchids (19).

## Results

### Phylogeny and Diversification.

**Fig. 1.**
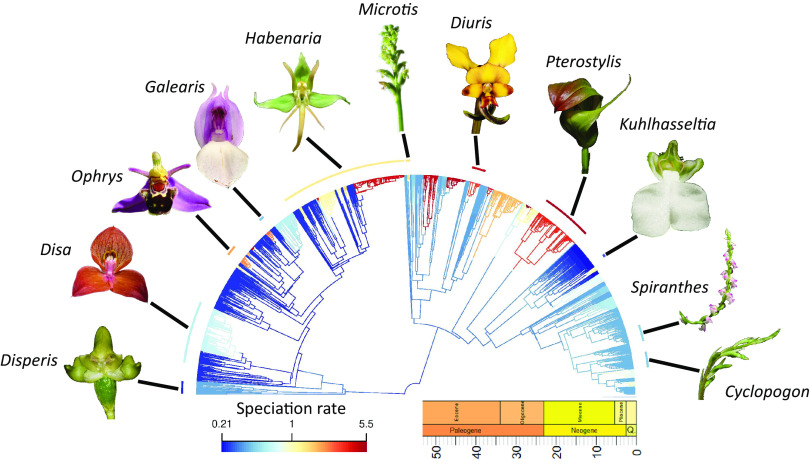
Time-calibrated phylogeny of 1,475 orchidoid taxa, visualized against the geological timescale. Branches are colored by speciation rates estimated with Bayesian Analysis of Macroevolutionary Mixtures. Orchidoid genera representing the diversity of form are associated with arc segments colored by their mean speciation rate. Images were sourced from Flickr (Creative Commons and modifications allowed).

Diversification rate is varied across Orchidoideae (Fig. 1). We inferred a maximum likelihood (ML) phylogeny of 1,475 taxa (29.5% of the 5,000 known species), using nine nucleotide loci mined from Genbank. The phylogeny is well supported, with ~62% of internal nodes having >70% bootstrap support (BS) and ~37% having >90% BS. Our tree is largely consilient with other recent estimates (19, 22). We used 15 secondary calibrations from previously inferred divergence estimates (19) to time-calibrate our tree with an uncorrelated relaxed clock in RelTime (23), which performs well in large empirical datasets (24, 25). The 15 secondary calibrations were themselves derived from plastome data and fossil constraints sampled across monocot orders in a Bayesian framework, using an uncorrelated, relaxed, lognormal clock (19).

We estimated speciation rates with Bayesian Analysis of Macroevolutionary Mixtures (BAMM), implemented with a reversible-jump Markov Chain Monte Carlo (MCMC) framework (26), accounting for incomplete sampling by specifying the fraction of known richness of each genus that was sampled (20). In all analyses with BAMM, we only considered speciation rate, not extinction rate, which avoids potentially unreliable parameter estimation (27–29) and is predicted to give more accurate estimates of speciation rate variability (30). We found 44-fold variation in the rate of speciation, with 36 core rate shifts in the best shift configuration, each of which indicated significant increases in speciation rate (overall rate heterogeneity: Bayes Factor >100). In the best shift configuration, rate shifts fell between ~26.21 Mya and the present day corresponding with a protracted period of climatic cooling in Earth’s history (31).

### Crossclade Consistency in the Consequences of Cooling.

**Fig. 2.**
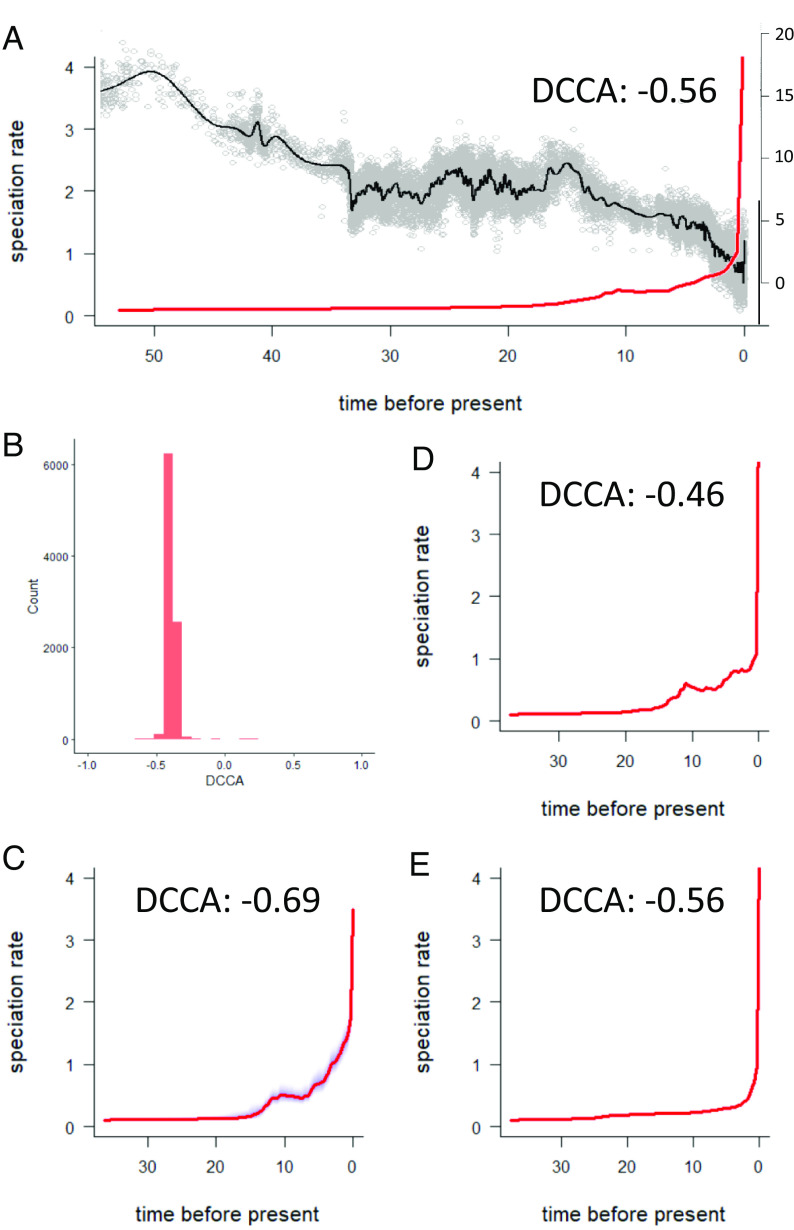
Consistency in the influence of global cooling on rapid speciation across Orchidoideae. The estimated historical speciation rate curve for the whole tree (red line with blue 95% CI) is plotted alongside the curve of estimated mean global paleotemperature (δ^18^O, black line) (*A*). A histogram of the DCCA coefficients used to infer the mean correlation coefficient across the whole tree (*B*). The estimated historical speciation rate curves of subclades Diurideae (*C*), Cranichideae (*D*), and Orchideae/Diseae (*E*) are reported with mean correlation coefficients indicated (all are consistently negative and significant at *P* < 0.0001).

**Fig. 3.**
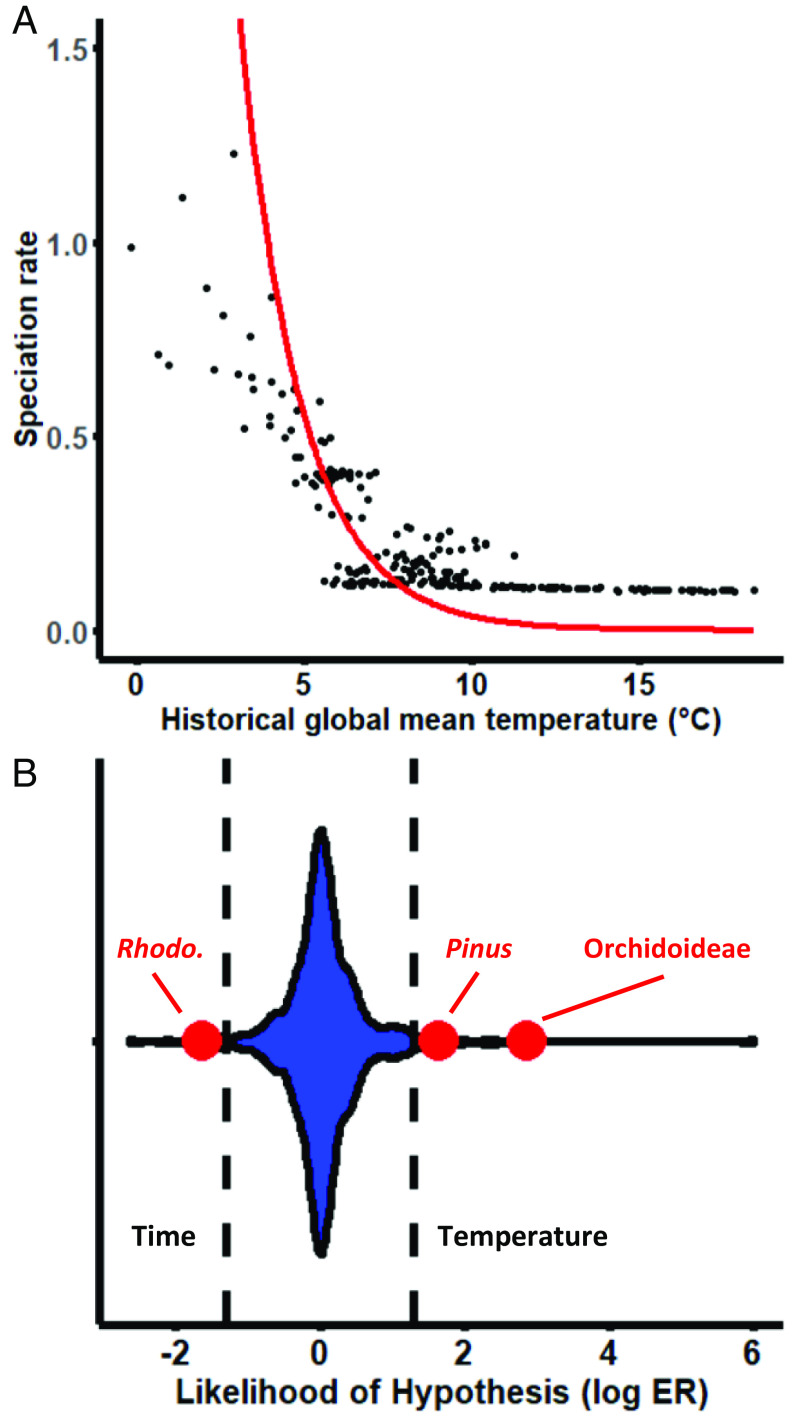
Strength of historic temperature on speciation rate. (*A*) The correlation between temperature and speciation rate (without estimating extinction) in terrestrial orchids is reported both for MCMC Bayesian analysis (BAMM, black dots) and likelihood-based exponential speciation model fitting (RPANDA, red curve), where speciation driven by global cooling is more likely than by time (ΔAICc = 248.6). (*B*) Evidential support for nested sets of temperature-dependent vs. time-dependent exponential speciation models is displayed as log evidence ratios, with hatched lines indicating taxa in which time or temperature is 20 times more likely. Temperature is 703.5 times more likely than time to drive speciation in the Orchidoideae (red dot on *Right*), one of the best-supported examples in comparison with 210 tetrapod groups (blue violin plot) and *Rhododendron* and *Pinus* plant groups (red dots on the *Left* and in the *Middle*, respectively).

To formally test the hypothesis that climatic cooling drove orchidoid speciation, we correlated 9,001 (10,000 minus 999 burn-in) realizations of the historical speciation curve with a reconstruction of Cenozoic δ^18^O (a proxy for mean global temperature) (29) and tested whether the distribution of correlation coefficients differed from zero. Across the subfamily, speciation rate had a strong and consistently negative association with Cenozoic δ^18^O [average of DCCA (detrended cross correlation analyses) correlation coefficients = −0.56, *P* < 0.0001; Fig. 2*B*]. Overall, we find strong evidence for a negative exponential correlation between estimated global paleoclimatic temperature data and mean BAMM-estimated speciation rates (r = −0.83, *P* < 0.0001; Fig. 3*A*). We find that global cooling is the most probable climatic driver, not CO_2_ or sea-level variation, which is important to consider because each is correlated (32) and have been shown to influence the speciation of other clades (e.g., refs. 10, 12, 33, and 34). Though the distributions of correlation coefficients were significantly different from zero, both atmospheric CO_2_ and sea level were more weakly correlated with speciation than that with temperature (average of DCCA correlation coefficients: CO_2_ = 0.29, *P* < 0.0001; sea level = −0.25, *P* < 0.0001). To assess potential for method artifacts of time calibration, we infer an alternative framework under penalized likelihood, which is known to perform more poorly and estimate different node ages than RelTime (23–25). Despite different node ages (t_1473_ = −61.4, *P* < 0.0001), we find that the node ages of RelTime and treePL frameworks are highly correlated (r = 0.97, *P* < 0.0001), and that both frameworks show strong relationships between global cooling and speciation rate (*SI Appendix*, Fig. S1 and S2).

Other studies have found that the influence of climate change varies between closely related clades (8, 9). But, we find crossclade consistency in the influence of climate change on speciation. Our DCCA reveals a negative relationship between the speciation curve and historical global temperature in each of the three major subclades (Cranichideae/Chloraeeae −0.46, *P* < 0.0001; Diurideae −0.69, *P* < 0.0001; and Orchideae/Diseae −0.56, *P* < 0.0001; Fig. 2).

### Chromosomal Number and Pollination Syndromes.

With data from the Chromosome Counts Database (35), we find that each of the major orchidoid subclades has more chromosomes than those of earlier diverging orchid subfamilies (Cranichideae/Chloraeeae mean = 21.52, t_256_ = 5.43, *P* < 0.0001; Diurideae mean = 21.45, t_106_ = 4.53, *P* < 0.0001; Orchideae/Diseae mean = 25.53, t_304_ = 9.31, *P* < 0.0001). And, as a subfamily, the orchidoids (mean = 23.96 ± 0.48 SE) have more chromosomes than those of both earlier diverging orchid subfamilies (Apostasioideae, Cypripedioideae, and Vanilloideae mean = 15.72 ± 0.83 SE; t_227_ = 8.77, *P* < 0.0001) and their more recently diverged, pollinia-containing sister group (Epidendroideae mean = 22.22 ± 0.20 SE; t_628_ = 3.37, *P* = 0.0008). Though these findings indicate that chromosome number has evolved independently within orchid subclades, the observed uniformity in chromosome number suggests it is unlikely that chromosomal count variation shaped diversification of terrestrial orchids.

We are unable to test for trait-driven influences on speciation, as the primary trait thought to drive orchidoid speciation, pollinia (packets of pollen), is found in all members (19). However, we can test for impacts of climate change in lineages with diverse pollination strategies. Consistent with findings across the subfamily, we find that the speciation curve of *Disa*, a genus characterized by a diversity of pollination syndromes with well-supported evidence of adaptive evolution (36, 37), is negatively correlated with global mean temperature (DCCA = −0.48, *P* < 0.0001).

### Temperature-, Not Time-, Dependent Speciation.

For taxa exhibiting steady increases in speciation rate with time, time is often found to be the most parsimonious explanation for diversification (13). Although the speciation rate of orchidoids increases up to the present (Fig. 2), we find substantially stronger support for models of global cooling than time. Both Bayesian MCMC (BAMM) and likelihood-based [RPANDA (38)] model–fitting approaches estimate similar relationships between historic temperature change and speciation rate (Fig. 3*A*). In tests with RPANDA, we find that models specifying exponential influences of historic temperature on speciation fitted patterns of orchid diversification substantially better than comparable time-dependent models, with or without estimating exponential extinction (ΔAICc = 13.1 and 248.6, respectively). Evidence ratios for temperature- and time-specific exponential speciation (estimated for models with no extinction, constant extinction, or exponential extinction) reveal that temperature-driven diversification is 703.5 times more likely than time dependence. To assess the extent to which the orchidoids represent an archetypal example of temperature-spurred diversification, we apply this methodology to 210 animal groups (13) and two other plant groups [*Rhododendron* (39) and *Pinus* (40)]. We find that the orchidoids represent one of the most convincing cases of temperature-driven speciation yet recorded (Fig. 3*B*).

### Lack of Temperature-Related Geographic Effects.

**Fig. 4.**
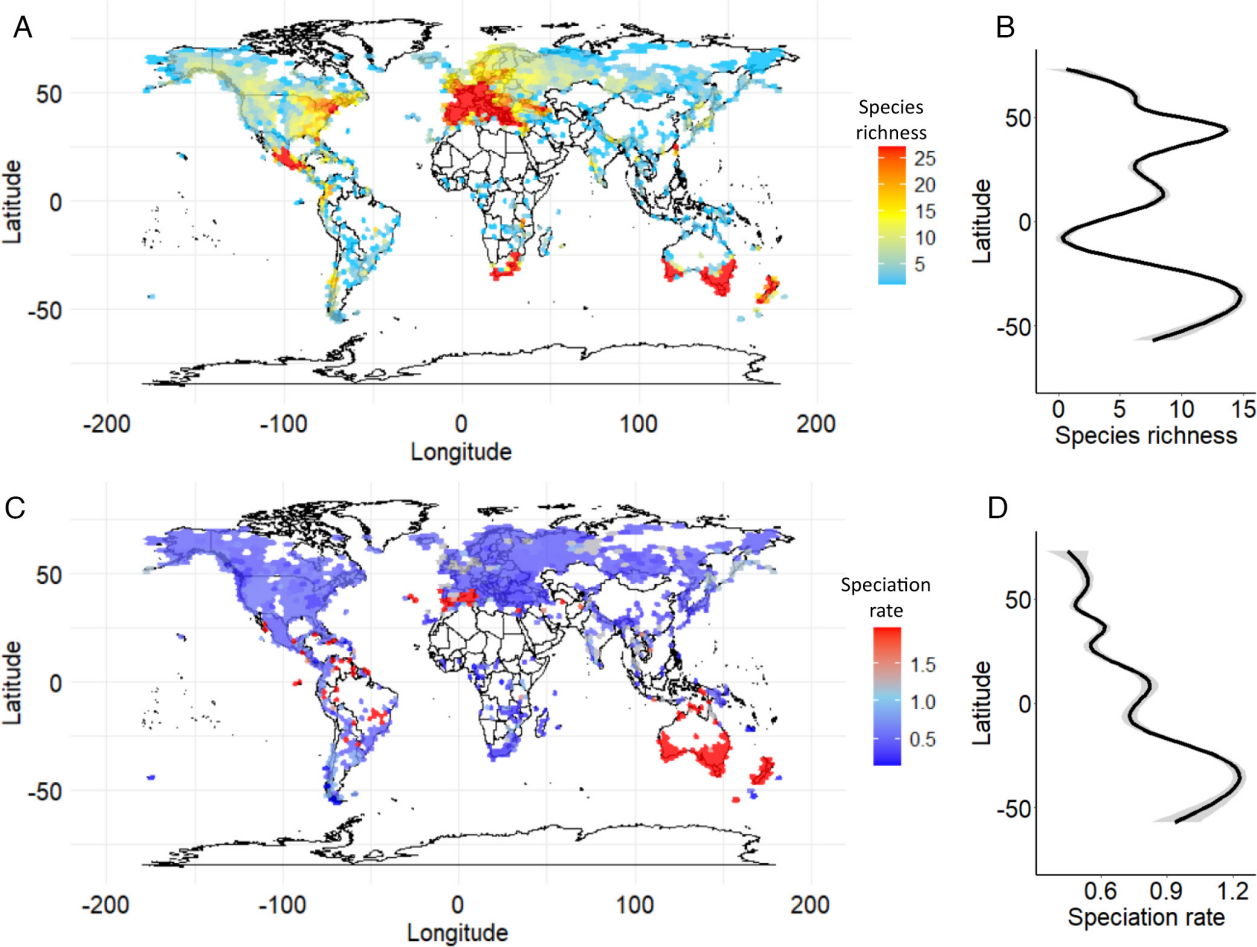
Global variation in orchidoid biodiversity. Species richness (*A*) and species richness by latitude (*B*) are calculated, respectively, from the number of orchidoid species represented in octagonal 200 m^2^ grid cells spanning the Earth and the average number of species in occupied grid cells by latitude fit to a generalized additive model. Speciation rate (*C*) and speciation rate by latitude (*D*) were modeled by BAMM, respectively, within each grid cell and averaged across occupied grid cells by latitude fit to a generalized additive model.

It has long been held that the tropics are a “cradle” of diversity for plant and animal taxa (14, 15). However, previous simulations of global impacts of climate change on biodiversity reveal that speciation rate can appear to be geographically varied even when climate change is the central driver of that diversification (7, 41). Speciation rate appears to be geographically varied in orchidoids. Consistent with other recent studies of angiosperm diversification (10, 14, 17, 42, 43), we find that orchidoid speciation is generally faster where it is cooler. Maps based on ~2.5 million georeferenced occurrence records (44) show that the orchidoids are globally distributed with peaks of richness centered in temperate regions and a peak in speciation rate evident in temperate regions of the Southern Hemisphere (Fig. 4). Although we find no significant difference in speciation rate when defining the tropics by temperature (tropics = >18 °C, nontropics = <18 °C) (*SI Appendix*, Fig. S3 and Table S1), orchidoid speciation rate is significantly lower in the tropics than that in temperate regions when defining the tropics by either binarized latitude (−23.5 to 23.5°) or continuous latitude (*SI Appendix*, Figs. S4–S6 and Table S1). However, we find no evidence of a causal relationship between geography and speciation rate, paralleling the lack of a latitudinal gradient in speciation rate seen across terrestrial orchids (19). After accounting for phylogenetic pseudoreplication, there were no associations between latitude and speciation rate, whether by continuous temperature [STRAPP (STructured Rate Permutations on Phylogenies), *P* = 0.85; Es-Sim *P* = 0.91], binarized temperature (STRAPP, *P* = 0.83) (45), binarized latitude (STRAPP, *P* = 0.77), or continuous absolute latitude (Es-Sim, *P* = 0.12; STRAPP, *P* = 0.40) (46). This implies that while there is latitudinal variation in speciation rate, the origin of new orchidoid species was primarily driven by global cooling, which cannot be explained by geographic influences on speciation rate.

Previous work indicates that diversification of Orchidaceae can be promoted by cooler highland distributions (43). We find elevational variation in speciation rate (*SI Appendix*, Fig. S5); however, phylogenetic tests reveal no evidence of relationships between elevation and speciation rate (minimum elevation in described species range: STRAPP, *P* = 0.16; Es-Sim, *P* = 0.37; maximum elevation in described species range: STRAPP, *P* = 0.82; Es-Sim, *P* = 0.11).

### Worldwide Cooling-Driven Speciation.

Previous biogeographic research has divided orchid distribution into seven geographical regions, defined as North America, Neotropics, Eurasia, Africa, Southeast Asia, Australia, and Pacific (21, 47). We find that there are significant differences between mean speciation rates between some bioregions. In particular, Australia has significantly higher tip speciation rates compared with all other bioregions (Africa: 1.01; Australia: 3.21; Eurasia: 0.95; North America: 0.57; Neotropics: 1.62; Pacific: 2.67; and Southeast Asia: 0.81). However, as with latitude and elevation, there is no evidence for any causal relationship between speciation rate and bioregion (STRAPP, Kruskal–Wallis, *P* = 0.23, *SI Appendix*, Fig. S6 and Tables S2–S4). Instead, we find negative DCCA correlations between historic mean global temperature and speciation rate through time in every bioregion except the Pacific (Africa: −0.39; Australia: −0.52; Eurasia: −0.37; North America: −0.41; Neotropics: −0.36; Pacific: 0.35; and Southeast Asia: −0.47, *P* < 0.0001 within each bioregion).

**Fig. 5.**
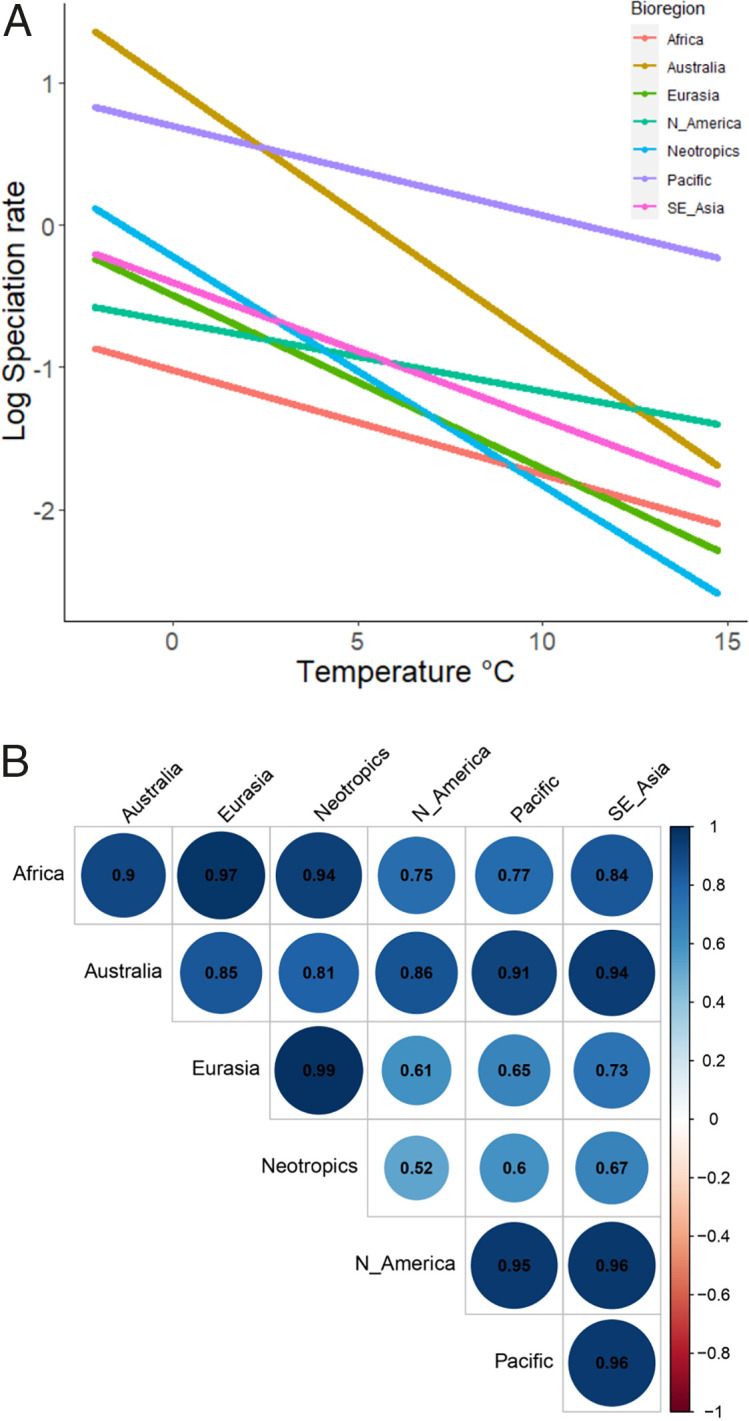
Geographic consistency in diversification driven by global cooling. Rate-through-temperature curves (*A*) of endemic lineages within each orchid bioregion were estimated by fitting exponential models to BAMM-derived speciation rate data covering the last 10 My. The correlation matrix of speciation rate through time curves (*B*) displays the degree to which diversification happened independently and simultaneously in endemic lineages across the Earth, from strong, +0.52 (light blue), to very strong, +0.99 (dark blue), correlations in each bioregion.

Our finding that climate cooling has global effects could be biased if taxa with high speciation rates are more likely to migrate between bioregions, or if responses to climate change occur in the same higher taxa predominating each bioregion. By focusing on species endemic to each bioregion, we confirm that speciation happened independently and simultaneously across the Earth. Although no bioregion contains just a single clade, each contains taxa that are phylogenetically clustered. This implies that terrestrial orchids have evolved independently in most bioregions, with the notable exception of the Pacific (Africa D = −0.33, n = 276; Australia D = −0.13, n = 241; Eurasia D = 0.16, n = 77; North America D = −0.07, n = 32; Neotropics D = −0.29, n = 236; Pacific D = 0.25, n = 30; Southeast Asia D = −0.22, n = 66) (48). Importantly, we find that speciation rate increases with global cooling in all the seven bioregions in independent fits of rate-through-temperature curves for data spanning the last 10 My (Fig. 5*A*). Shifts in speciation rate between bioregions are also positively temporally correlated (Fig. 5*B*), providing strong support for independent, contemporaneous speciation driven by global cooling.

### Diverse Regional Responses to Other Aspects of Climate Change.

It is unlikely that geographic consistency in the influence of global cooling results from insufficient power to detect variation. Consistent with previous work (9–12), we find evidence of bioregion-specific variation of climate change on speciation (Table 1). Global atmospheric CO_2_ is positively correlated with speciation in each bioregion except North America. By contrast, there is little evidence that global sea-level variation influences speciation in any bioregion, despite being coupled with global temperature change (Table 1). Thus, though some aspects of mean global climate change are geographically varied, our study shows that global cooling drove speciation across the Earth.

**Table 1.** Geographic variation in the consequences of mean global atmospheric CO_2_ and sea level

Bioregion	Atmospheric CO_2_	Sea level
Africa	0.33**	−0.12
Australia	0.42**	−0.28
Eurasia	0.40**	−0.18
Neotropics	0.40**	−0.18
North America	0.15	−0.13
Pacific	0.17*	−0.27
Southeast Asia	0.44**	−0.18

DCCA correlations between speciation rate and climate indices within each bioregion are reported with significance indicated (*P < 0.05, **P < 0.001).

## Discussion

The need for a global conservation strategy is almost universally acknowledged (49–53), and a better understanding of the forces shaping biodiversity will be necessary in order to achieve this objective (6, 54–60). Unfortunately, it has proven difficult to disentangle the impacts of local climate from the historical consequences of global climate change and the passage of time. Previous models predict that global climate change has a role in shaping patterns of standing biodiversity and biodiversity change (7, 61). However, the notion that climate change is responsible for the global distribution of biodiversity has been largely dismissed. Our study shows that although global cooling is the major driver of the rise of terrestrial orchids, their species richness also has marked biogeographical variation. We find that the ecological variables influencing changes in biodiversity can be obscured unless they are analyzed in an evolutionary context. This finding has fundamental and applied implications.

Orchidoid biodiversity evolved in a manner counter to the predictions of many theories of diversification. Although Orchidoideae were not part of Darwin’s abominable mystery, since they originated well after the initial radiation of angiosperms (1–5), our findings provide a clear example in which gradual diversification is not supported, and recent radiations are driven by climate change. As speciation in terrestrial orchids is driven by global cooling, rather than warming, our findings run counter to classic explanations of rapid ecological niche filling (61) and the metabolic theory of biodiversity (62). We find no support for cradles of orchid diversity, nor evidence for highlands- or tropics-driven speciation (14, 17, 42, 43). Moreover, previous work reports great variation in the effects of climate upon diversification rate, even for closely related lineages (8–10) and in closely similar or proximate ecoregions (11, 12). Here, by contrast, we find that historic temperature changes exerted a consistent influence on biodiversity change. It is likely that there are undiagnosed roles for climate change in other major radiations. Recent macroecological work suggests that global cooling may have a systemic role in angiosperm speciation (59). The variation we found in three lineages of angiosperms highlights the need for a large-scale meta-analysis of climatic effects on diversification across major plant groups, similar to that recently undertaken for tetrapods (13). Unfortunately, for many highly speciose plant clades, such as cacti, *Euphorbia,* and other succulents, there are scant paleoclimatic data on aridity and other abiotic factors likely to be of relevance. We predict that climate change will be associated with speciation in the largely epiphytic epidendroid orchids because, as in their orchidoid sister clade, they exhibit recent explosive radiation, a consistent pollinia phenotype, and higher chromosome counts than those of earlier diverging orchid clades.

There is growing evidence of the influence of climate change on evolution. Recent work shows that the famous diversification of cichlid fish was driven by a temporally complex mixture of tectonic activity, climate change, biotic resource flow, and interspecific hybridization (63, 64). Rapid evolution in response to climate change is well established in particular species of Darwin’s finches and Anolis lizards (65, 66). The persistence of ancient alleles in these groups (67, 68) is consistent with the presence of high genetic variability early in island colonization; but, it is also consistent with colonization events followed by climate change–induced genetic variability. Although our study cannot differentiate between these models of evolution, it does highlight the need for detailed, location-specific records of historic climatic variation, as we found bioregion-specific effects of global average CO_2_ on speciation.

### How Does Climate Change Drive Evolution?

It is not clear how global cooling drives diversification. Milankovitch (orbital) cycles change the exposure to annual solar energy in predictable ways, but responses on Earth, including biotic changes, tectonic activity, and climate change, can be varied (69). A role for tectonics is unlikely, as it is expected to have localized geographic impacts (69). The global expansion of C_4_ grasslands which peaked 4 to 8 Ma (70–73) could have contributed to more recent orchidoid speciation by creating new habitats.

The relatively short-term oscillations in global temperature occurring during longer-term trends of global cooling may have a critical role in accelerating orchidoid speciation; the evidence for global temperature oscillations is restricted to recent time periods (74). While the underlying mechanism is likely to be the same across Orchidoideae, we note that the Australian bioregion has the highest inferred speciation rate and the strongest evidence of relationships between global temperature and speciation rate. As a consequence of the onset of the Antarctic Circumpolar Current approximately 30 Mya (75), the Australian continent separated from Antarctica and moved north, resulting in dramatic cycles of cooling and drying across Australia (76, 77). These repeated cooling cycles may have stimulated higher speciation rates relative to those in other bioregions, the signature of which is preserved in the high tip speciation rates seen today. Overall, although speciation rates appear to be higher in the Southern Hemisphere (Fig. 4*D*), we find little statistical evidence for the influence of latitude.

Global cooling could disrupt gene flow, thereby stimulating diversification, by influencing the physiology of plants, symbiotic fungi, and/or pollinators. Terrestrial orchids are sensitive to temperature variability (78, 79). The symbiotic mycorrhizal fungi of orchids provide cold-assisted improvements in germination (80), but also contribute to population subdivision (81). The tight coevolution between orchidoids and their pollinators first identified by Darwin (82, 83) may also provide a mechanism for rapidly generating barriers to gene flow. Indeed, pollination can be reduced by the influences of climate change on plant phenology and by the physiological limitations of pollinating insects in colder temperatures (84).

Although we find consistent responses to global cooling across orchid bioregions and the three major tribes of terrestrial orchids, this does not preclude the possibility of differential speciation in response to global cooling. Our analysis suggests that in the major tribes, Orchideae/Diseae, Cranichideae, and Diurideae, increased speciation might be concentrated in *Habenaria* in the first, in *Pterostylis* in the second, and across the clade in the third. In Diurideae and *Pterostylis,* it is highly plausible that climate deterioration could have provided an advantage for deceit pollination, which may have contributed to rapid speciation in those groups.

Climatic cooling could also drive rapid speciation by increasing genetic variability. Several studies show that biotic and abiotic factors, particularly temperature, can alter meiotic recombination in ways which can accelerate adaptation (85, 86). Major changes in genome size and GC content are associated with orchidoid diversification (87, 88). Although we find that each of the major orchidoid subclades has more chromosomes than those of earlier evolving orchid taxa, without evidence of cooling-induced changes in chromosomal number, the more parsimonious hypothesis is that high chromosomal numbers evolved early. Our finding of a well-resolved orchidoid phylogeny with high speciation rates is consistent with speciation occurring simultaneously in reproductively isolated populations, perhaps as a consequence of climate-mediated stress (89), and, occasionally, allotetraploidy events (90).

### Implications for Conservation.

This research provides a large and well-supported case study of the long-term impacts of climate change on biodiversity. Preserving hot spots of diversity has been a central tenet of conservation, with substantial theoretical support (91). However, these hot spots have not typically been identified by accounting for the ecological processes that generate the biodiversity we are trying to conserve. If global speciation from climate change is common and unrelated to localized biodiversity, then conserving areas with low species richness may be just as important for preserving evolutionary potential.

Resolving whether climate change stimulates adaptive radiation through stronger selection, population fragmentation or increased genetic variability has implications for conservation. There has been substantial interest in whether plants have sufficient standing genetic variation to respond to predicted gradual global warming (92). But, the history of the Earth is marked by catastrophic shifts in climate (93), while climate change can impose strong breeding system selection (e.g., refs. 94 and 95). The most relevant question for conservation could therefore be whether plant species have sufficient capacity to generate genetic variation in response to climate-induced stress. Our finding that global cooling independently stimulated the formation of the thousands of orchidoid species within a short period (30 Ma) demonstrates that changes in climate can have predictable influences on speciation. Our study establishes terrestrial orchids as an excellent model system for understanding how interactions between climate change and physiological traits drive rapid speciation.

## Methods

### Supermatrix Assembly.

We mined Genbank for Orchidoideae sequences using the OneTwoTree pipeline (96), filtering intraspecific varieties, hybrids, and open nomenclature. We corrected nomenclature against The Plant List (www.theplantlist.org), which reduces the impact of poor taxonomic assignment within Genbank. OneTwoTree clustered sequences into orthologous groups, which we inspected and edited by reclustering partial sequences with full sequences. Although this could have resulted in unreliable alignments, OneTwoTree selected the longest sequence for every species, and we visually inspected resulting alignments. We downloaded Orchid outgroup sequences from Genbank and aligned these with ingroup sequences using Mafft -add (97). We trimmed unreliably aligned positions with trimAl -gappyout command (98) and concatenated alignments into a supermatrix using Alignment Manipulation And Summary (AMAS) (99). Finally, we removed taxa with identical sequences, since these are known to create short terminal branches that distort phylogenetic inference (100).

### Phylogenetic Reconstruction.

We produced a time-calibrated phylogeny in three steps. After an initial ML search with 1,000 BS replicates using RAxML V8 (101), we identified and removed taxa exhibiting rogue behavior in the BS replicates using RogueNaRok (102). This is an especially important procedure when using published sequences, reducing the impact of "chimeric taxa" created when sequences are misidentified and concatenated, and which would therefore contain conflicting phylogenetic signal (103). We performed another ML search on this pruned dataset in order to obtain the final molecular phylogeny used for our divergence time analysis. ML searches were performed in the Cyberinfrastructure for Phylogenetic Research (104). Each ML search used 1,000 BS replicates to assess branch support and applied an individual GTR model of nucleotide substitution to each locus partition. We enforced the monophyly of three clades in both searches: tribes Orchideae/Diseae, tribe Diurideae, and tribe Cranichideae (see ref. 20), in order to improve the likelihood calculation, as in other large phylogenies (105). Because large datasets pose a computational burden for molecular dating methods that rely on Bayesian MCMC sampling, we calibrated the ML phylogeny against geological time using the relaxed-clock ML method RelTime (23–25). Orchids are poorly represented in the fossil record, with only one orchidoid fossil assigned with certainty [to subtribe Goodyerinae (106, 107)]. Instead, we used robust secondary calibrations (19), applying minimum and maximum ages for 15 major clades as uniform constraints, according to the upper and lower bounds of 95% CIs.

In order to assess the robustness of our method of time calibration, we reconstructed an alternate framework using penalized likelihood with treePL (108). The alternate framework employed the same node age constraints as RelTime. An initial treePL run was used to prime parameters for the final analysis, in which multiple smoothing parameters were tested and crossvalidated. We plotted the distribution of node ages in both frameworks and confirmed similarity with a Pearson’s product–moment correlation (0.97, *P* < 0.0001).

### Speciation Analysis.

We reconstructed speciation history using the BAMM framework (26), sampling four MCMC chains of 25 million generations every 2,500th generation. We set priors with the R package BAMMtools (109) and used a conservative prior of one rate shift. Rate shifts were permitted only in clades of more than five species, to improve convergence. Genus-level sampling fractions were derived from a recent checklist (20) and were specified in order to account for heterogeneous sampling across clades. We excluded the first 10% of generations as burn-in, and assessed convergence with the R package Coda (110), confirming that the effective sample size of each parameter was >400. We ignored reconstructions of extinction rate, which are known to be unreliable when modeled from phylogenies containing only extant taxa (27). We plotted speciation rates through time against temperature curves for both frameworks and found that the relationship between global cooling and speciation rate was robust in both frameworks (*SI Appendix*, Fig. S2). The results are presented for the RelTime framework, which is known to be more accurate than penalized likelihood (24, 25).

We fitted time- and temperature-dependent ML models of diversification to the orchidoid phylogeny using the R package R: Phylogenetic ANalyses of DiversificAtion (RPANDA) (38), using code adapted from ref. 111. Initially, we fitted 18 models starting from the simplest to models with increasing complexity. For each of time and temperature, these included constant speciation with no extinction; linear speciation with no, constant, linear, or exponential extinction; and exponential speciation with no, constant, linear, or exponential extinction. We excluded constant speciation models from further analysis because of their poor explanatory power. We excluded linear speciation and extinction models because they are known to be problematic (112) and are biased against finding temperature-driven speciation. For example, we find that 80% (66/82) of the taxa with best support (delta AIC < 4) for temperature-driven speciation and extinction in a large study of tetrapods (13) exhibit nonlinear model fits over relevant temperature ranges which give a false impression of better fit to models of time than temperature. For direct comparisons of influences between temperature and time, we ultimately fitted 3 models (exponential speciation with no, constant, or exponential extinction) for both temperature and time to the orchidoid phylogeny by ML. We calculated the corrected Akaike Information Criterion (AICc), the ΔAICc, and the Akaike weight (AICω) to assess likelihood support. We calculated the ΔAICc between specific models to test support for temperature and time and plotted the best-performing model against temperature and BAMM-estimated speciation rates. To compare the relative support for temperature vs. time across the three models, we calculated evidence ratios, estimated as Σ AICω temperature models/Σ AICω time models, for orchidoids in addition to published RPANDA parameters from 210 phylogenies (13) and estimated parameters for two published plant phylogenies [*Rhododendron* (39) and *Pinus* (40)]. The distribution of the relative support for time- vs. temperature-driven diversification across the 213 phylogenies was displayed as violin plots of log-transformed evidence ratios to improve visualization.

### DCCA.

We performed DCCA with the 9,001 post-burn in realizations of the speciation curve in R, using code from Davis et al. (8). Briefly, we Tukey-smoothed paleoclimatic proxies and interpolated values to the times recorded in the speciation rate curves. We calculated correlation coefficients between each of the 9,001 post burn-in speciation curves and paleoclimatic proxy and plotted the distribution. A −1 indicates perfect negative correlation, 0 indicates no correlation, and +1 indicates perfect positive correlation (8). DCCAs were chosen over traditional correlation methods such as Pearson’s product–moment correlation, because the time series are autocorrelated over shorter timespans. A Wilcoxon rank-sum test assessed significance of the distribution from the null hypothesis of zero (no correlation). Note, we did not include the monotypic tribe Codonorchideae in the analysis of crossclade consistency. Paleoclimatic proxies of global mean temperature, atmospheric CO_2_, and sea-level were sourced from Zachos et al. (31), Bergmann et al. (113), and Miller et al. (114), respectively.

### Chromosomal Counts.

We downloaded available chromosomal counts for all Orchidaceae from the Chromosome Count Database (35), using the median value where multiple counts were reported for a species.

### Biogeographical Analyses.

We downloaded georeferenced occurrences from the Global Biodiversity Information Facility (GBIF) (44) using rgbif (115) and cleaned coordinates with the R package CoordinateCleaner (116). We removed coordinates with uncertainty >10 km, and those near capital cities, biodiversity institutions, those with equal longitude and latitude values, and within seas, resulting in >2.5 million cleaned records. To test for causal influences of spatial climatic variation, we used STRAPP (45), Es-Sim (46), and phylogenetic signal calculated for binary traits with the D statistic (48) in the R package caper (117). In our analysis of tropical and nontropical speciation rates, two definitions of tropical and nontropical were used: a geographical definition and a temperature definition. Species were defined as tropical geographically if >50% of their occurrence localities lay within ±23.5° of the equator. Species were defined as tropical according to temperature if the year-round monthly temperature at >50% of their occurrence localities exceeded 18 °C (118, 119). To calculate the latter, we retrieved data of mean annual temperature (1981 to 2010) from CHELSA V2.1 (120). These binary trait-dependent speciation analyses were performed with STRAPP, using 1,000 permutations, and significance was assessed with a Mann–Whitney test. In our analysis of speciation rates of major bioregions, we sorted taxa into seven bioregions defined by Givnish et al. (21) (Africa, Australia, Europe, North America, the Pacific, and Southeast Asia), excluding taxa present in >1 bioregion. We conducted another STRAPP test, using 1,000 permutations, and assessed significance with a Kruskal–Wallis test. We used BAMMtools to estimate mean speciation rate through time curves for each bioregion and tested temporal relationships between bioregions with Pearson’s correlations. In our analysis of elevation-dependent speciation, we acquired minimum and maximum elevation from the Internet Orchid Species Photo Encyclopaedia (http://orchidspecies.com/) and assessed elevation-dependent speciation with STRAPP and Es-Sim, with both tests implementing 1,000 permutations. Es-Sim uses a Pearson’s test to assess significance, and we used a Spearman test in the STRAPP analysis. Elevation data from orchidspecies.com are likely to be partially qualitative, but elevation data in GBIF were poor. When performing DCCAs for regional speciation, we used all 9,001 post burn-in speciation curves when investigating the role of global cooling, but used mean speciation rate when correlating regions with each other, and with atmospheric CO_2_ and sea level. For comparability, within bioregion, DCCA correlations between atmospheric CO_2_ and sea level were estimated for the same time period of 37.8 Mya to the present day. We visualized variation in the relationship between speciation rate and temperature across bioregions in the most recent 10 Ma, the timeframe encompassing the most dramatic radiations. To do this, we fitted exponential models between historical speciation rate of bioregion-endemic species and paleotemperature (d^18^O) (31) and plotted the outcome.

## Supplementary Material

Appendix 01 (PDF)

## Data Availability

Nucleotide accessions, phylogenetic trees, input and output files for analyses have been deposited in Data Dryad (121). All study data are included in Dryad and/or *SI Appendix*.
