# Supplementary material for: Speciation across the Earth driven by global cooling in terrestrial orchids
Source: Proc Natl Acad Sci U S A. 2023 Jul 10;120(29):e2102408120. doi: 10.1073/pnas.2102408120 (PMC10629580; doi:10.1073/pnas.2102408120)
Supplement: Supplementary file 1 — Appendix 01 (PDF) [file pnas.2102408120.sapp.pdf]

PNAS

Supporting Information for Speciation across the Earth driven by global cooling in terrestrial Orchids

Jamie B. Thompson, Katie E. Davis, Harry O. Dodd, Matthew A. Wills, Nicholas K. Priest

Corresponding authors: Jamie B. Thompson and Nicholas K. Priest
Email: jbt38@bath.ac.uk, np253@bath.ac.uk

This PDF file includes:

Supporting text
Figures S1 to S8
Tables S1 to S4
SI References

Supplementary Results

Extended results for speciation rate analyses. Full results for speciation rate analyses showing geographic effects on tip speciation rates.

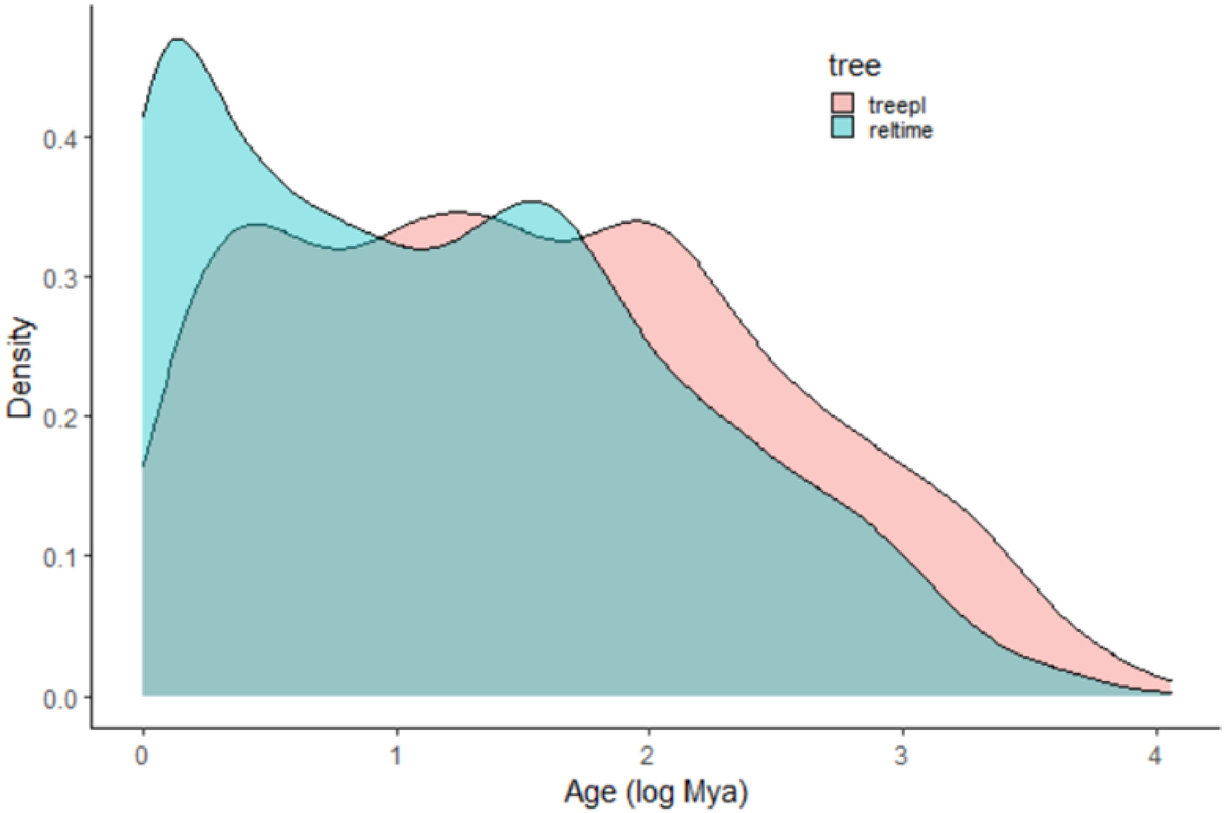

Fig. 1: Distributions of node ages estimated under Maximum Likelihood with RelTime, and Penalised Likelihood with treePL. Densities of log transformed ages are plotted with RelTime estimates in blue shading and treePL estimates in pink.

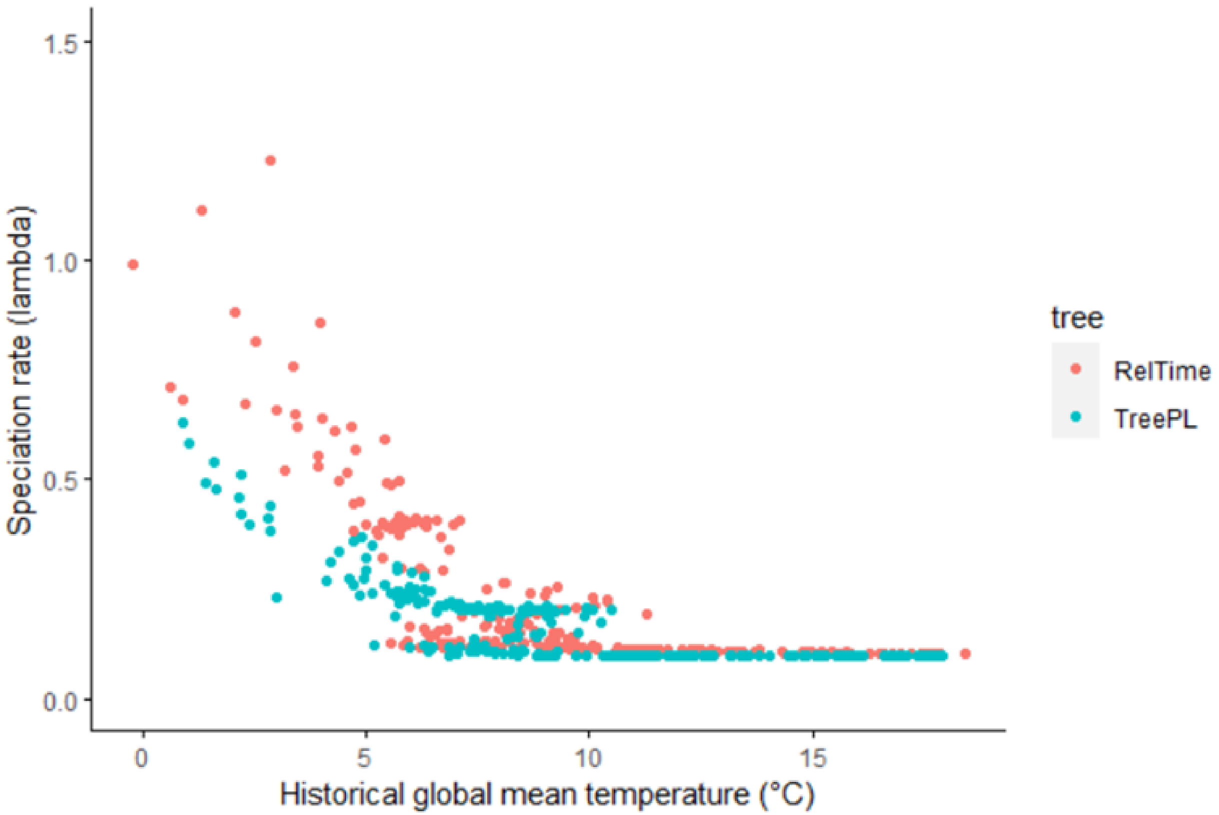

Fig. S2: Consistency in the relationship between speciation and global cooling regardless of divergence estimation method. Historical global mean temperature is plotted against BAMM-estimated speciation rates through time estimated with the RelTime framework (pink dots) and TreePL framework (blue dots).

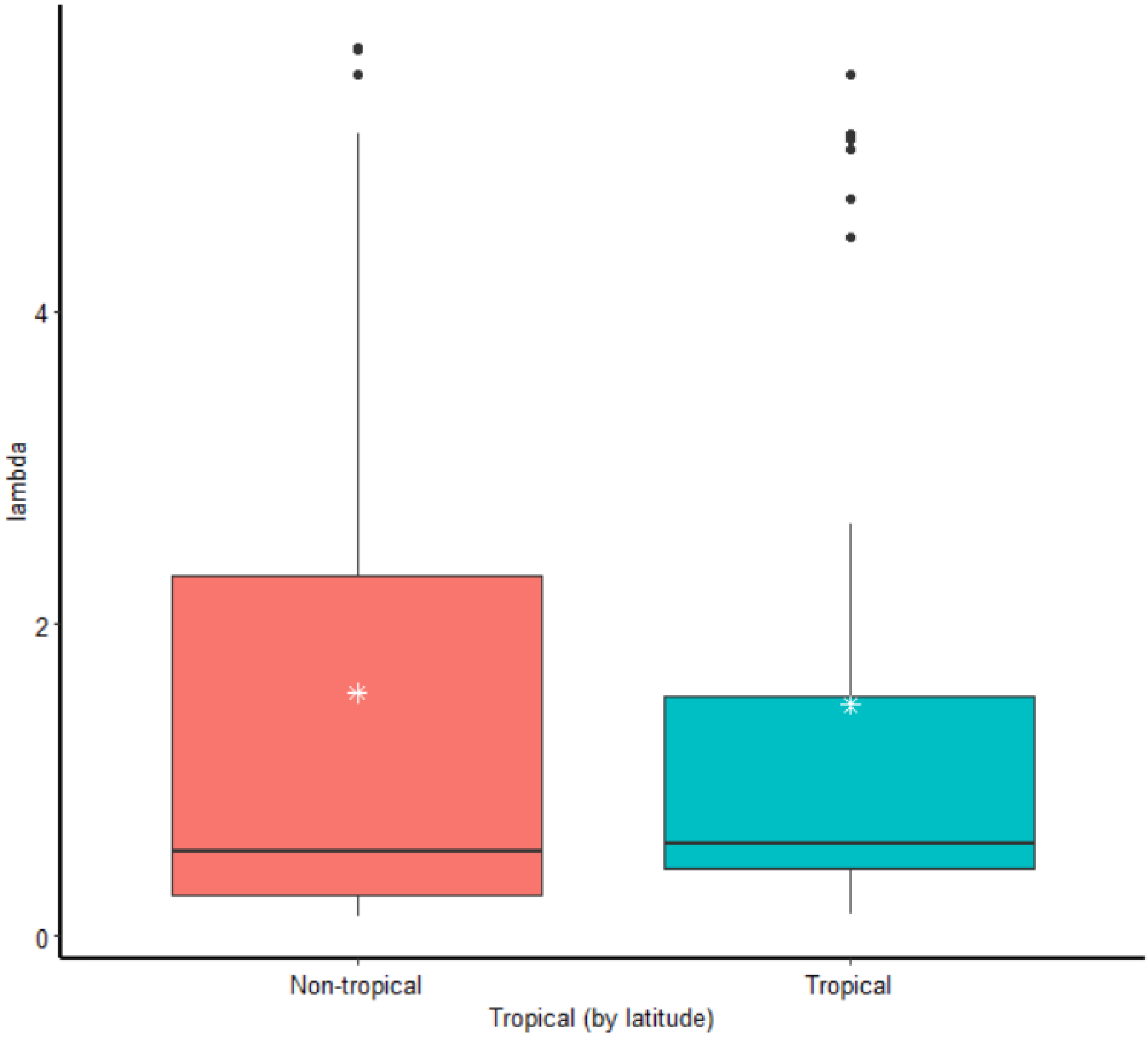

Fig. S3. Tip speciation rates (species/million years) compared between tropical and non-tropical taxa where tropics are defined by temperature =>18°C, and non-tropics are defined as temperature= <18°C. Pink shading indicates tropical taxa and turquoise shading indicates non-tropical taxa. The upper and lower bounds of the shaded boxes represent the first and third quartiles, the bold line represents the median value for speciation rate. Speciation rates were modelled in BAMM (1) and extracted with the R package BAMMtools (2).

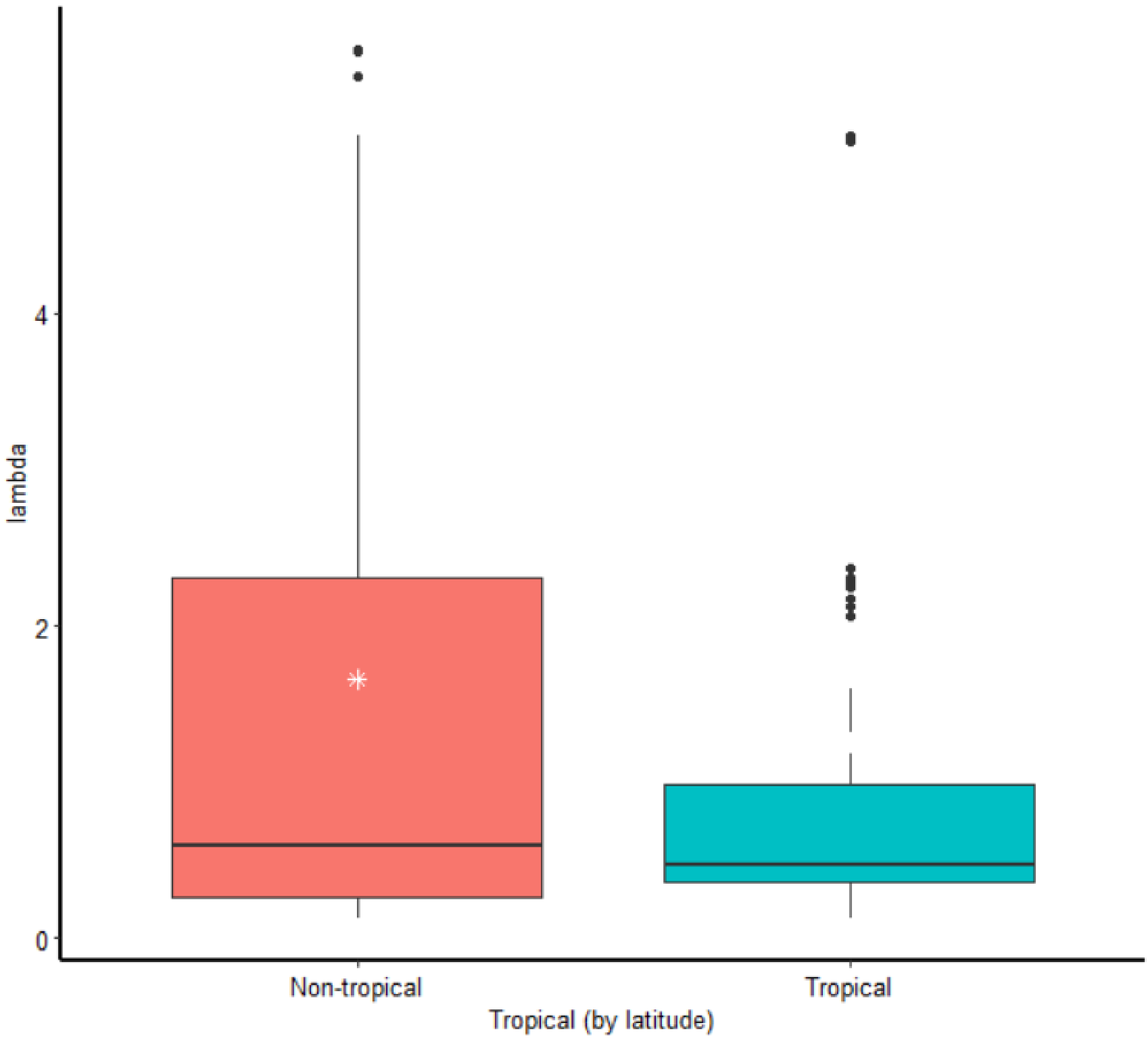

Fig. S4. Tip speciation rates (species/million years) compared between tropical and non-tropical taxa where tropics are defined by binarised latitude (-23.5 to 23.5 degrees). Pink shading indicates tropical taxa and turquoise shading indicates non-tropical taxa. The upper and lower bounds of the shaded boxes represent the first and third quartiles, the bold line represents the median value for speciation rate. Speciation rates were modelled in BAMM (1) and extracted with the R package BAMMtools (2).

Table S1. Mean tip speciation rates (species/million years) and p-values for binarised temperature and latitude. Bold text indicates p-value < 0.01. While mean speciation rates are lower in tropical taxa for both data partitions, the difference is only significant for binarised latitude. Note that the standard deviation is large for all four analyses. Speciation rates were modelled in BAMM (1).

	Mean speciation rate: Tropical	Standard deviation: Tropical	Mean speciation rate: Non-tropical	Standard deviation: Non-tropical	P-value
Binary temperature	1.4821	1.7778	1.5608	1.7977	0.5431
Binary latitude	**1.2508**	**1.6721**	**1.6572**	**1.8260**	**0.0005**

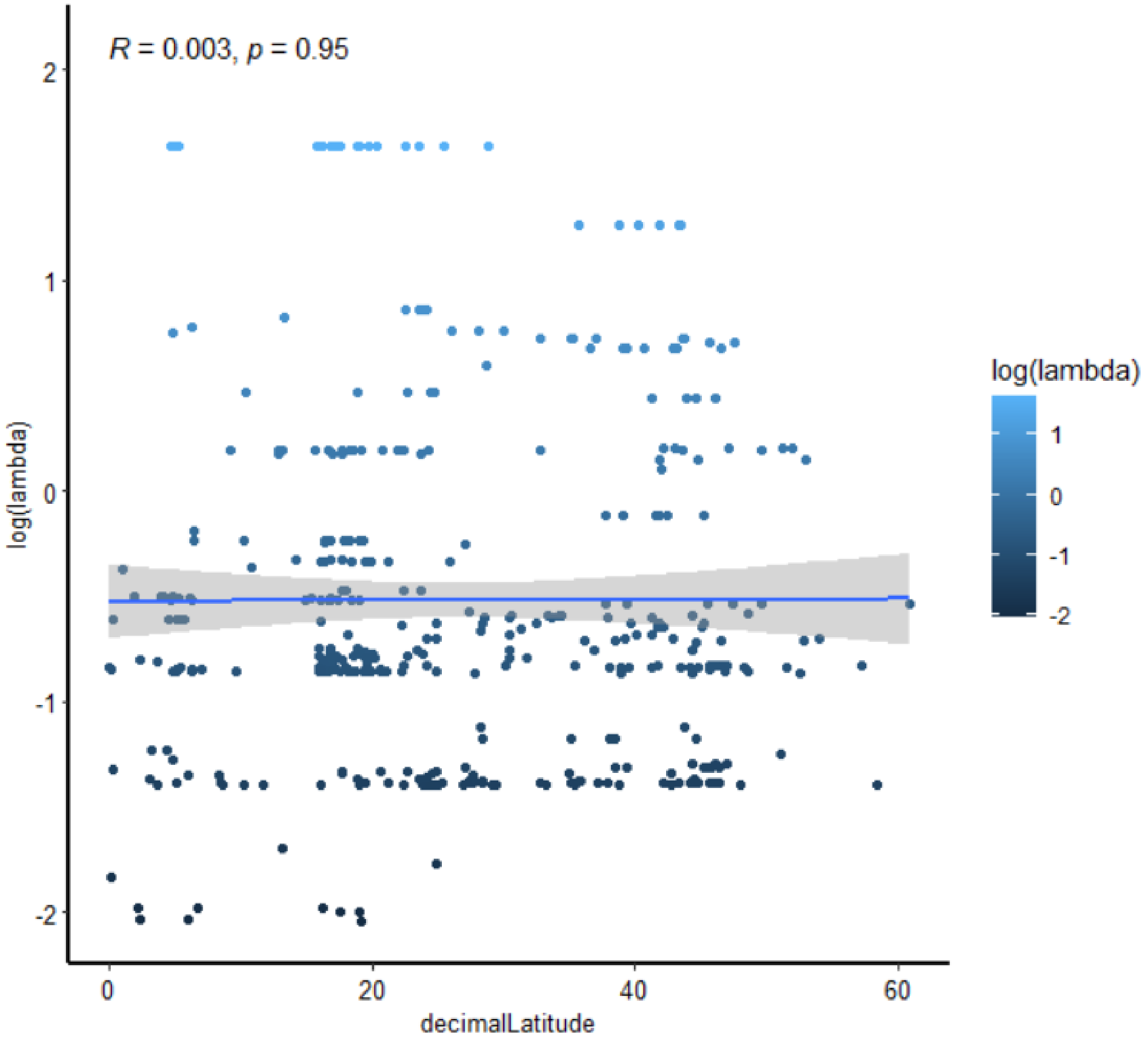

Fig. S5. Tip speciation rates (species/million years on a log scale) plotted against latitude in the northern hemisphere as a continuous variable, r=0.003, p-value=0.95. Speciation rates were modelled in BAMM (1) and extracted with the R package BAMMtools (2).

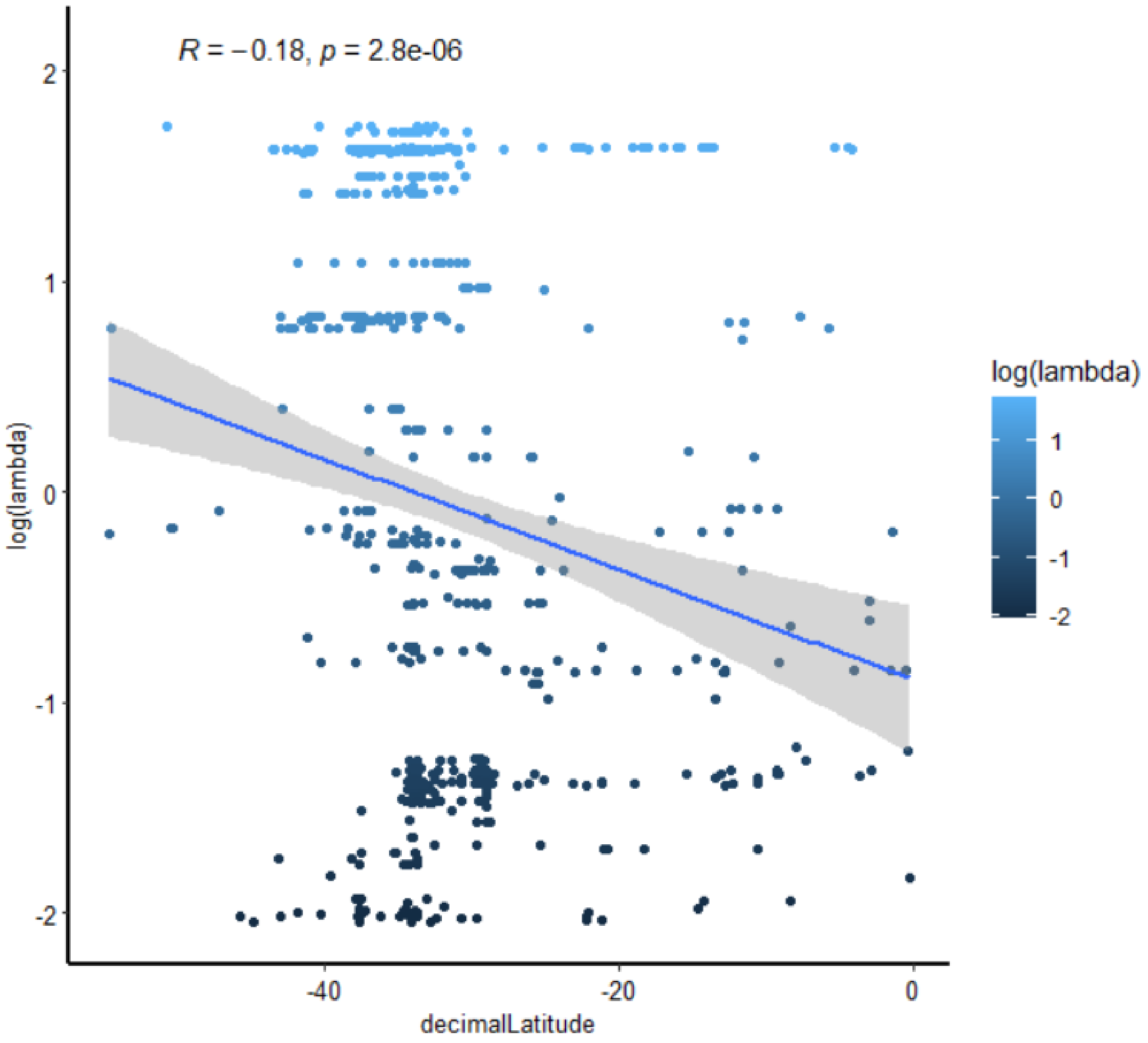

Fig. S6. Tip speciation rates (species/million years on a log scale) plotted against latitude in the southern hemisphere as a continuous variable, r=-0.18, p-value=2.6e^{-06}. Rates modelled in BAMM (1) and extracted with the R package BAMMtools (2).

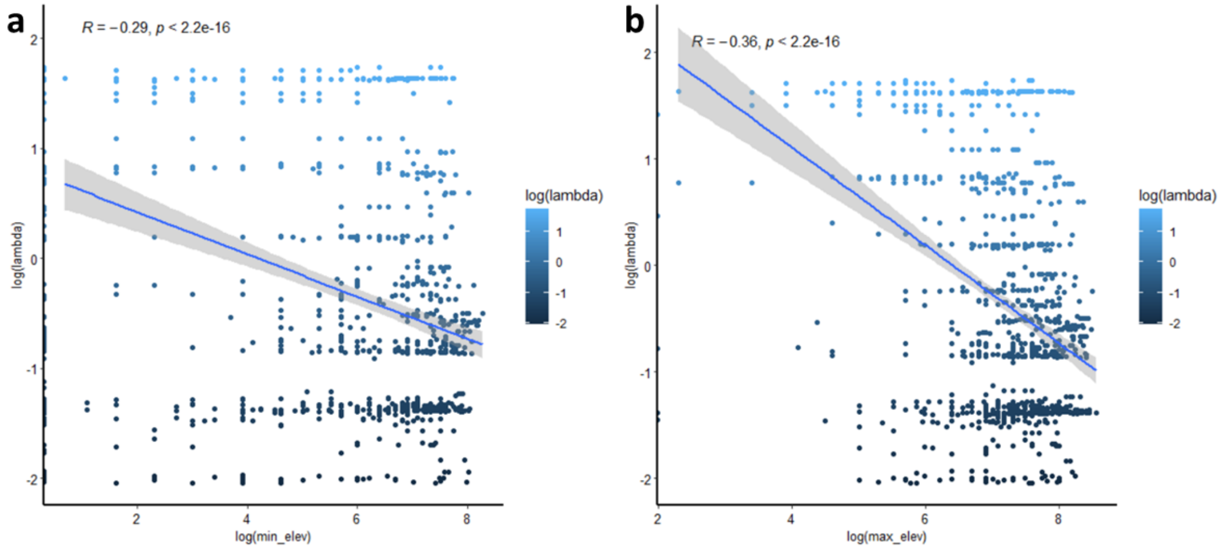

Fig. S7. Tip speciation rates (species/million years on a log scale) plotted against minimum elevation (metres) as a continuous variable (**a**), r=-0.29, p-value=2.2e^{-16}, and maximum elevation (**b**), r=-0.36, p-value=2.2e^{-16}. Rates modelled in BAMM (1) and extracted with the R package BAMMtools (2).

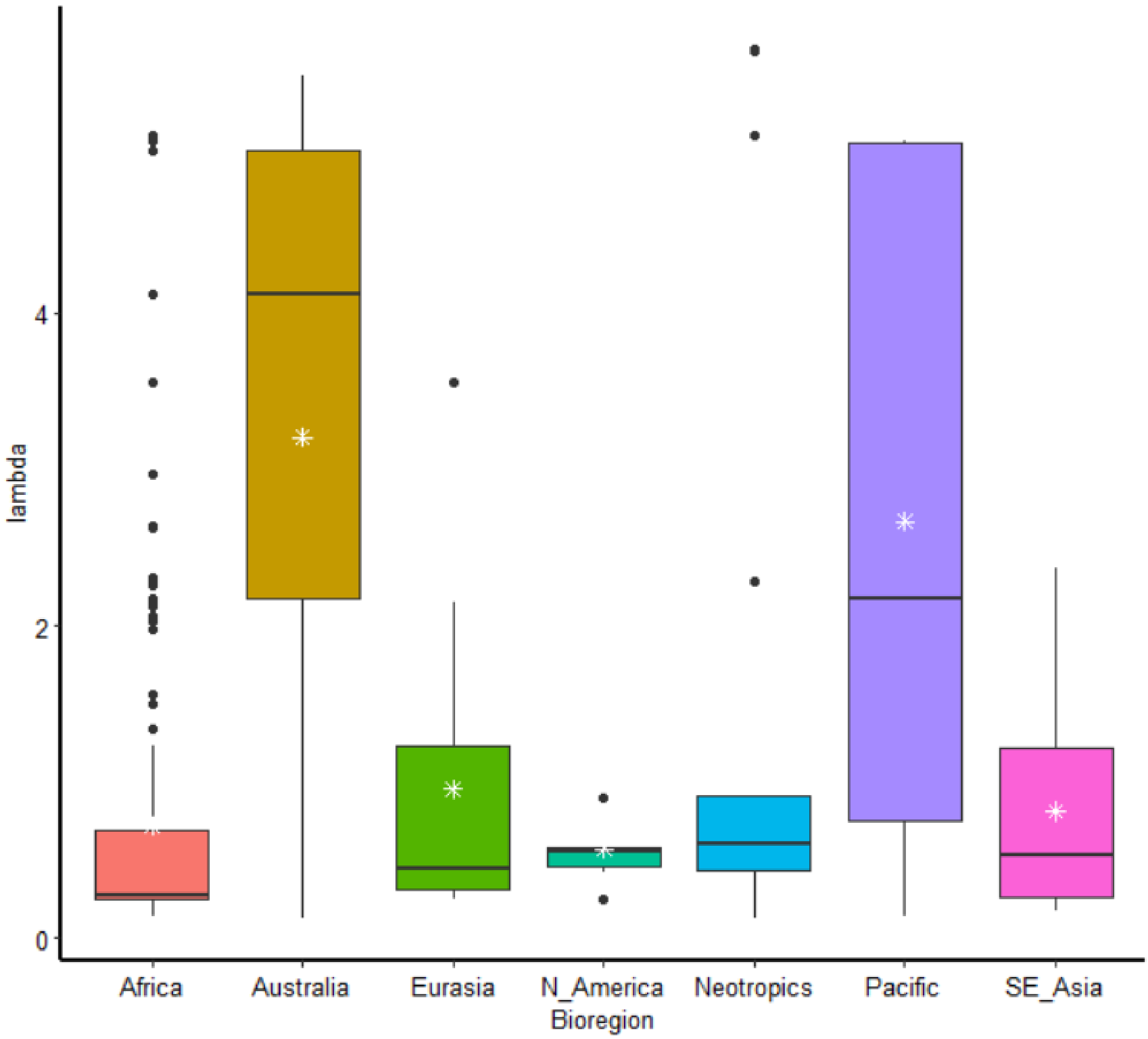

Fig. S8. Tip speciation rates (species/million years) across major orchid bioregions. From left to right, blue shading = Africa bioregion, pink shading = Australia bioregion, green shading = Eurasia bioregion, red shading = Neotropics bioregion, yellow shading - North America bioregion, turquoise shading = Pacific bioregion and purple shading = Southeast Asia bioregion. The upper and lower bounds of the shaded boxes represent the first and third quartiles, the bold line represents the median value for speciation rate. Rates modelled in BAMM (1) and extracted with the R package BAMMtools (2).

Table S2. Mean tip speciation rates (species/million years) across bioregions. Speciation rates modelled in BAMM (1).

**Bioregion**	**Mean speciation rate**	**Standard deviation**
Africa	1.009	0.723
Australia	3.205	1.884
Eurasia	0.953	0.931
Neotropics	1.619	2.000
North America	0.570	0.177
Pacific	2.668	1.977
Southeast Asia	0.811	0.651

Table S3. Pairwise comparisons of mean tip speciation rates (species/million years) across bioregions. Bold text indicates p-value < 0.01 after Bonferroni correction.

Bioregion	P-value
**Australia-Africa**	**4.96e-78**
Eurasia-Africa	0.217
**Neotropics-Africa**	**5.85e-13**
North America-Africa	0.578
**Pacific-Africa**	**1.10e-11**
Southeast Asia-Africa	0.658
**Eurasia-Australia**	**6.15e-29**
**Neotropics-Australia**	**2.75e-29**
**North America-Australia**	**4.47e-20**
Pacific-Australia	0.064
**Southeast Asia-Australia**	**5.01e-29**
**Neotropics-Eurasia**	**7.21e-04**
North America-Eurasia	0.224
**Pacific-Eurasia**	**1.22e-07**
Southeast Asia-Eurasia	0.571
**North America-Neotropics**	**2.08e-04**
**Pacific-Neotropics**	**3.11e-04**
**Southeast Asia-Neotropics**	**1.11e-04**
**Pacific-North America**	**4.29e-08**
Southeast Asia-North America	0.455
**Southeast Asia-Pacific**	**2.21e-08**

Table S4. Formal testing for causality between geographic variables and tip speciation rates. P-values are reported for two methods used, STRAPP (3) and Es-Sim (4). None of the results were significant.

	**STRAPP p-value**	**Es-Sim p-value**
Binary temperature	0.83	NA
Binary latitude	0.77	NA
Continuous raw latitude	0.29	0.26
Continuous absolute latitude	0.40	0.12
Elevation minimum	0.16	0.27
Elevation maximum	0.08	0.37
Bioregion	0.23	NA

SI References

1. D.L. Rabosky. Automatic detection of key innovations, rate shifts, and diversity- dependence on phylogenetic trees. *PLoS ONE* **9**: e89543 (2014).
2. D.L. Rabosky et al. BAMM tools: an R package for the analysis of evolutionary dynamics on phylogenetic trees. *Methods Ecol. Evol.* **5**, 701–707 (2014).
3. D.L. Rabosky & H. Huang. A robust semi-parametric test for detecting trait-dependent diversification. Syst. Biol., 65, 181-193 (2016).
4. M.G. Harvey & D.L. Rabosky. Continuous traits and speciation rates: alternatives to state-dependent diversification models. Methods Ecol. Evol. 9, 984-993 (2018).
